# Disability pension and mortality in individuals with specific somatic and mental disorders: examining differences between refugees and Swedish-born individuals

**DOI:** 10.1136/jech-2019-213436

**Published:** 2021-01-20

**Authors:** Magnus Helgesson, Syed Rahman, Fredrik Saboonchi, Ellenor Mittendorfer Rutz

**Affiliations:** 1 Division of Insurance Medicine, Department of Clinical Neuroscience, Karolinska Institutet, SE-171 77 Stockholm, Sweden; 2 Department of Health Sciences, The Swedish Red Cross University, College, SE-102 15 Stockholm, Sweden

**Keywords:** disability, epidemiology, mortality, public health, social epidemiology

## Abstract

**Background:**

More than half a million refugees have arrived to Sweden during the last decade. The aim was to investigate differences between refugees and Swedish-born individuals regarding different specific somatic and mental disorders, and subsequent disability pension and mortality.

**Methods:**

All refugees (n=239 742) and Swedish-born individuals (n=4 133 898), aged 19–60 years, resident in Sweden on 31st of December in 2009 were included in this population-based prospective cohort study. Data from six nationwide Swedish registers were linked by the unique anonymised identification number. HRs with 95% CIs were computed for disability pension and mortality 2010–2013 by Cox regression models.

**Results:**

Compared with their Swedish-born counterparts with mental or somatic diagnoses, refugees with these diagnoses had a higher risk of subsequent disability pension and a lower risk of mortality. Highest estimates for disability pension were seen for refugees with neoplasm (HR: 1.72; 95% CI: 1.56 to 1.91), musculoskeletal disorders (HR: 1.57; 95% CI: 1.47 to 1.67), diseases of the circulatory system (HR: 1.33; 95% CI: 1.22 to 1.45), depressive disorders (HR: 1.31; 95% CI: 1.21 to 1.41) and diabetes mellitus (HR: 1.30; 95% CI: 1.15 to 1.47). The risk of mortality was lowest for refugees with regard to bipolar disorders (HR: 0.37; 95% CI: 0.16 to 0.82), post-traumatic stress disorder (HR: 0.37; 95% CI: 0.25 to 0.54) and least pronounced in regard to neoplasm (HR: 0.69; 95% CI: 0.61 to 0.77) compared with Swedish-born with similar disorders.

**Conclusion:**

Refugees have a generally higher risk of disability pension compared with Swedish-born with specific somatic and mental disorders. Despite this, refugees with all specific disorders have lower risk estimates of mortality, probably due to a healthy selection. The higher risk of disability pension might therefore be due to other causes besides poor health.

## Introduction

Sweden has, like several other European countries, become a multicultural society due to increasing global migration. Of note, more than half a million refugees arrived to Sweden during the last decade, and many of them have experienced health-impacting traumatic life events and bereavement in their home countries and during the flight.^1^ This development puts refugees’ health issues on the receiving societies’ public health agenda. In the 1980s and 1990s, most of the refugees originated from Iran, Iraq, Turkey and former Yugoslavia; and from the beginning of the 2000s, also many refugees from northeast Africa arrived to Sweden.^2^ Most refugees arrived to Sweden by themselves, but about 11% of all refugees during the period from 1980 to 2007 arrived to Sweden as resettled refugees on a quota negotiated by the Swedish government and the United Nations High Commissioner for Refugees.^2^

A higher prevalence of somatic diseases, for example circulatory, respiratory and digestive disorders, compared with the host population, has been found among migrant subgroups.^3–6^ Moreover, the prevalence of mental disorders is high among refugees.^7^ This combined vulnerability may start a downward spiral of worsening health, leading to socioeconomic hardship after arrival in the host country.^8^ In addition, poor accessibility to healthcare, less propensity and fear of seeking healthcare due to structural, cultural and lingual barriers among refugees might, on the one hand, worsen the course of the illness, and on the other hand add to the difficulties of finding a position at the labour market.^9–12^ The link between premature exit from the labour market due to health reasons, that is, disability pension and subsequent premature death, is well described in the literature.^13 14^ Still, these two outcomes might be differently driven by adverse health, poor socioeconomic conditions and negative health behaviour.^15 16^ As most other studies, both generally and from our research group, have a focus on mental disorders,^7 12 17–19^ studies that also elucidate the role of specific somatic disorders in the pathways to mortality and disability pension among refugees are warranted. There are to the best of our knowledge very few longitudinal studies related to specific mental disorders and no studies that are related to specific somatic disorders and subsequent adverse social and health outcomes, measured as disability pension and mortality among a refugee population.

Many studies have paradoxically found that immigrants in general have a mortality advantage compared with individuals in the host population.^20–23^ Given that immigrants form a heterogeneous group, within-group differences in mortality risk might be expected. Refugees, in particular, might be at higher risk for adverse health and social outcomes due to the high likelihood of traumatic and life-threatening experiences, and the lack of access to healthcare during the often long journeys to Western host countries. The aim was to study the associations between commonly occurring somatic and mental disorders and subsequent disability pension and mortality among refugees, and to investigate if there were differences in such associations between refugees and Swedish-born individuals.

## Methods

### Study population

All refugees and Swedish-born individuals between 19 and 60 years of age, who were resident in Sweden on 31st of December 2009, were included in the study base (n=4 376 055). Refugees with no data on reason for settlement to Sweden (n=307) or contradicting information on reasons of migration (n=2108) were excluded. The final population comprised of 4 373 640 individuals, of which 239 742 were refugees and 4 133 898 were Swedish-born individuals. In analyses regarding disability pension as outcome measure, individuals with baseline disability pension (n=314 922) were excluded.

Refugees were in this study defined as: (1) individuals who received a residence permit in Sweden according to the rules in the United Nations Geneva Convention of Refugees from year 1951, (2) individuals who received a residence permit in Sweden due to humanitarian reasons, or (3) individuals who, due to need of protection, including resettled refugees, received a residence permit in Sweden. A sensitivity analysis, which compared each of these groups separately with Swedish-born individuals, was performed and showed similar results.

### Register data

Data from different nationwide Swedish registers were linked by the unique anonymised identification number, from the following agencies:

1. Statistics Sweden: *Longitudinal integration database for health insurance and labour market studies, available since 1990*; sex, age, educational level, family situation, type of living area, country of birth, income from work and unemployment. *Longitudinal database for integration studies, available since 1997*: reason for settlement in Sweden.
2. Social Insurance Agency: *Micro-data for analyses of social insurance;* date and duration of sickness absence and disability pension.
3. The National Board of Health and Welfare: *National Patient Register (NPR*): main and secondary diagnoses for mental and somatic disorders from inpatient and specialised outpatient healthcare; *Cause of Death Register*: date of mortality; and the *Prescribed Drug Register:* prescription of diabetes medication according to the Anatomical Therapeutic Chemical Classification (ATC) code: A10.

### Variables and diagnoses

#### Exposure

Main or secondary diagnoses from inpatient and specialised outpatient healthcare or specific prescribed medication for somatic and mental disorders, as exposure, were divided into dichotomised variables of following groups of diagnoses, recorded according to the International Classification of Disorders version 10 (ICD-10)^24^ : (1) infectious and parasitic diseases (A00–B99), (2) neoplasm (C00–D48), (3) diabetes mellitus (E10–E14 or a record of diabetes medication according to ATC code A10),^25^ (4) diseases of the circulatory system (I00–I99), (5) diseases of the respiratory system (J00–J99), (6) diseases of the digestive system (K00–K93), (7) diseases of the musculoskeletal system and connective tissues (M00–M99), (8) diseases of the nervous system (G00–G99), (9) other somatic disorders (E00–E09, E15–E90, H00–H99, L00–99, N00–T99), (10) anxiety disorders (F40, F41, F42), (11) bipolar disorder (F31), (12) depressive disorders (F32, F33), (13) post-traumatic stress disorder (F43.1), (14) other stress-related disorders (F43.0, F43.2, F43.8, F43.9) and (15) other mental disorders (other F-diagnoses).

#### Outcomes

The cohort was followed 2010–2013 with regard to: (1) disability pension and (2) mortality. In Sweden, all residents between 19 and 64 years of age can be granted disability pension if their work capacity is permanently or in the long term reduced due to disease or injury. Basic levels of disability pension can be granted without previous income from work. As opposed to the eligibility criteria for unemployment benefits, the requirements for being granted disability pension include work incapacity related to a disease. For this reason, the decision to receive disability pension is based on medical assessments and is often (not always) preceded by long periods of sickness absence. While being granted sickness absence requires income from work, basic levels of disability pension can be granted without previous income from work. The regulation regarding disability pension was tightened in 2008, and only individuals 30 years of age and above with an expected life-long disability could be granted permanent disability pension in Sweden. However, young adults (19–29 years of age) can still be granted temporary disability pension if their work capacity is reduced or compulsory school education not completed.

#### Covariates

The covariates included in the analyses were (1) *sociodemographic factors:* sex, age, educational level, type of living area and family situation (all variables were measured on 31st December 2009; see table 1 for categorisations); (2) *work-related factors:* income from work and unemployment, measured in 2009 (see table 1 for categorisations) and (3) *health-related factors:* a record of a main or secondary diagnosis from specialised healthcare due to mental (ICD-10: F00–F99) or somatic disorders (ICD-10: A00–E99, G00–T99) during 2006–2009 and sickness absence during 2009 (see table 1 for categorisations). Missing values in any covariate were treated as a separate category in the adjusted analyses.

**Table 1:** Covariates at baseline and outcome during the follow-up among refugees and Swedish-born individuals registered in Sweden in 2009 (n=4 373 640)

	Refugees	Swedish-born individuals
	n (%)	n (%)
**Total number**	239 742 (100)	4 133 898 (100)
**Sociodemographic factors** *		
Sex		
Men	138 798 (57.9)	2 116 306 (51.2)
Women	100 944 (42.1)	2 017 592 (48.8)
Age (years)		
16–24	35 112 (14.7)	621 697 (15.0)
25–34	57 668 (24.1)	888 316 (21.5)
35–44	62 365 (26.0)	1 048 791 (25.4)
45–54	64 437 (26.9)	984 653 (23.8)
55–64	20 160 (8.4)	590 441 (14.3)
Educational level		
Low (0–9 years)	60 317 (25.2)	494 445 (12.0)
Medium (10–12 years)	98 302 (41.0)	2 128 041 (51.5)
High (>12 years)	70 858 (29.6)	1 491 839 (36.1)
Missing information	10 265 (4.3)	19 573 (0.5)
Type of living area†		
Big cities	115 942 (48.4)	1 475 443 (35.7)
Medium-sized cities	87 056 (36.3)	1 493 126 (36.1)
Small cities/villages	36 744 (15.3)	1 165 329 (28.2)
Family situation		
Married/cohabiting without children living at home	20 228 (8.4)	440 134 (10.7)
Married/cohabiting with children living at home	107 230 (44.7)	1 573 743 (38.1)
Single‡ without children living at home	84 040 (35.1)	1 661 526 (40.2)
Single with children living at home	21 050 (8.8)	282 990 (6.9)
Children (=20 years old) living at home	7192 (3.0)	175 503 (4.3)
Missing information	<10	<10
Country of birth		
Eritrea	4517 (1.9)	–
Ethiopia	4645 (1.9)	–
Somalia	12 905 (5.4)	–
Other countries in Africa	5933 (2.5)	–
Afghanistan	4969 (2.1)	–
Iran	27 355 (11.4)	–
Iraq	49 156 (20.5)	–
Syria	6539 (2.7)	–
Other countries in Asia	27 871 (11.6)	–
Chile	8058 (3.4)	–
Other countries in South America	3567 (1.5)	–
Former Yugoslavia	70 433 (29.4)	–
Other (including persons with missing information)	13 794 (5.8)	–
**Work-related factors** *§*		
Income from work**¶**		
Yes	122 817 (51.2)	3 313 944 (80.2)
No	116 925 (48.8)	819 954 (19.8)
Unemployment**		
No unemployment	176 380 (73.6)	3 672 075 (88.8)
Unemployment 1–180 days	40 515 (16.9)	364 336 (8.8)
Unemployment >180 days	22 847 (9.5)	97 487 (2.4)
**Health-related factors**		
Sickness absence**		
No sickness absence	225 348 (94.0)	3 851 056 (93.2)
Sickness absence 1–90 days	11 729 (4.9)	239 157 (5.8)
Sickness absence >90 days	2665 (1.1)	43 685 (1.1)
Somatic disorders		
Infectious and parasitic diseases	17 225 (7.2)	207 086 (5.0)
Neoplasm	12 987 (5.4)	257 732 (6.2)
Diabetes mellitus	10 173 (4.2)	97 274 (2.4)
Diseases of the circulatory system	15 576 (6.5)	232 827 (5.6)
Diseases of the respiratory system	17 259 (7.2)	242 170 (5.9)
Diseases of the digestive system	26 308 (11.0)	330 367 (8.0)
Diseases of the musculoskeletal system	34 801 (14.5)	518 724 (12.6)
Disorders of the nervous system	11 810 (4.9)	181 693 (4.4)
Other somatic disorders	126 174 (52.6)	1 969 345 (47.6)
Mental disorders		
Anxiety disorder	7715 (3.2)	107 436 (2.6)
Bipolar disorder	627 (0.3)	20 809 (0.5)
Depression	9807 (4.1)	105 158 (2.5)
Post-traumatic stress disorder	4902 (2.0)	5090 (0.1)
Other stress-related disorders	5381 (2.2)	48 663 (1.2)
Other mental disorders	8984 (3.8)	163 686 (4.0)
Outcomes during the follow-up (2010–2013)		
Disability pension	3804 (1.75)	29 820 (0.78)
Mortality	1310 (0.55)	30 041 (0.73)

*All sociodemographic factors were measured on 31st of December in 2009.

†Big cities: Stockholm, Gothenburg and Malmo; medium-sized cities: cities with more than 90 000 inhabitants within 30 km distance from the centre of the city; small cities/villages.

‡Single includes divorced, separated or widowed.

§All work-related factors were measured during year 2009.

¶Categorical variable (1) income from work over 100 SEK, (2) no income from work.

**Unemployment and sickness absence was measured during 2009.

### Statistics

First, after checking that the assumption of proportional hazards was met, crude and multivariate Cox regression models were used. HRs with 95% CIs were computed in relation to disability pension and mortality among Swedish-born individuals and refugees with and without somatic/mental disorders, with Swedish-born individuals without any somatic/mental disorders as the reference group. Thereafter, crude and multivariate adjusted HRs for diagnosis specific somatic/mental disorders (at ICD-10 group level) were calculated for refugees compared with Swedish-born with the same diagnoses. With regard to analyses of general somatic/mental disorders, the analyses were adjusted in three steps: model 1, sociodemographic factors; model 2, in addition adjusted for work-related factors, that is, income from work and unemployment; and model 3, in addition adjusted for health-related factors, that is, sickness absence and occurrence of a main or a secondary diagnosis of a mental/somatic disorder between 2006 and 2009. In addition, sex-stratified analyses were performed regarding pooled data of somatic and mental disorders. With regard to analyses on specific somatic/mental disorders, the analyses were only adjusted in one step, as in model 3 above. All analyses were performed with SAS statistical software, V.9.4.

## Results

### Characteristics

There was a higher proportion of men among refugees (58%) compared with Swedish-born individuals (51%) (table 1). Most of the refugees arrived from former Yugoslavia (29%), Iraq (20%), Iran (11%) and other countries in Asia (12%). Refugees had in general a lower educational level and lived to a higher extent in big cities compared with Swedish-born individuals. A substantially lower proportion of refugees (51%) had income from work compared with Swedish-born individuals (80%). Refugees had also a higher prevalence of long-term unemployment, rather equal prevalence of long-term sickness absence, as well as a higher prevalence of both mental (except for bipolar disorder) and somatic (except for neoplasm) disorders during 2006–2009 compared with Swedish-born individuals.

### Disability pension

#### Somatic disorders

In the crude model, refugees with somatic disorders had almost seven times higher risk for disability pension compared with Swedish-born individuals without somatic disorders (table 2). The risk estimates were also two times as high compared with Swedish-born individuals with somatic disorders. In the final model, refugees with somatic disorders (HR: 2.7; 95% CI: 2.5 to 2.8) had just slightly higher risk estimates of disability pension compared with Swedish-born individuals with somatic disorders (HR: 2.0; 95% CI: 2.0 to 2.1). No sex differences in these associations were found.

**Table 2:** HRs and 95% CIs of disability pension and mortality among refugees and Swedish-born individuals with or without a somatic disorder, resident in Sweden in 2009; follow-up 2010–2013

	n (%)*	Crude	Model 1†	Model 2 ‡	Model 3§
			HR (95% CI)		
**Disability pension (n=4 058 718)**					
Swedish-born individuals, no somatic disorders	5609 (0.35)	1	1	1	1
Refugees, no somatic disorders	634 (0.76)	2.23 (2.06 to 2.42)	2.16 (1.98 to 2.35)	1.28 (1.17 to 1.39)	1.40 (1.29 to 1.52)
Swedish-born individuals, with somatic disorders	24 211 (1.09)	3.15 (3.06 to 3.24)	3.11 (3.02 to 3.21)	3.09 (3.00 to 3.18)	2.04 (1.98 to 2.10)
Refugees, with somatic disorders	3170 (2.37)	6.92 (6.63 to 7.23)	6.85 (6.55 to 7.17)	3.97 (3.79 to 4.15)	2.65 (2.53 to 2.77)
**Mortality (n=4 373 640)**					
Swedish-born individuals, no somatic disorders	7010 (0.42)	1	1	1	1
Refugees, no somatic disorders	267 (0.30)	0.74 (0.66 to 0.84)	0.79 (0.70 to 0.89)	0.56 (0.49 to 0.63)	0.58 (0.51 to 0.65)
Swedish-born individuals, with somatic disorders	23 031 (0.94)	2.26 (2.20 to 2.33)	2.23 (2.17 to 2.29)	2.08 (2.03 to 2.14)	1.70 (1.65 to 1.75)
Refugees, with somatic disorders	1043 (0.69)	1.66 (1.55 to 1.77)	1.72 (1.61 to 1.84)	1.15 (1.08 to 1.23)	0.93 (0.87 to 1.00)

*Number of individuals with the outcome during the follow-up period.

†Adjusted for sociodemographic factors, that is, sex, age, educational level, type of living area and family situation, measured on 31st of December 2009.

‡Like model 1 and additionally adjusted for work-related factors, that is, income from work and unemployment, measured during 2009.

§Like model 2 and additionally adjusted for sickness absence during 2009 and occurrence of a main or a secondary diagnosis of a mental disorder 2006–2009 according to International Classification of Disorders version 10: F00–F99.

When the analyses were stratified by specific somatic disorders, the crude model showed that refugees with all subgroups of specific somatic disorders had a significantly higher risk for disability pension compared with Swedish-born individuals with these disorders (table 3). In the full model, however, there was a substantially higher risk for refugees compared with Swedish-born individuals only for those with neoplasm, diseases of the musculoskeletal system, diabetes mellitus, diseases of the circulatory systems and within the group with other somatic disorders (HR range: 1.3–1.7). Foremost work-related and health-related factors altered the risk estimates of disability pension.

**Table 3:** HRs and 95% CIs of disability pension and mortality among refugees with specific somatic disorders compared with Swedish-born individuals with similar somatic disorders, resident in Sweden in 2009; follow-up 2010–2013

	Disability pension (n=4 058 718)			Mortality (n=4 373 640)		
	n (%)*	Crude	Adjusted†	n (%)*	Crude	Adjusted†
**Infectious and parasitic diseases**						
Swedish-born individuals	3028 (1.68)	1	1	4469 (2.16)	1	1
Refugees	429 (2.87)	1.72 (1.56 to 1.91)	1.14 (1.02 to 1.27)	224 (1.30)	0.60 (0.53 to 0.69)	0.52 (0.45 to 0.60)
**Neoplasm**						
Swedish-born individuals	3267 (1.43)	1	1	6663 (2.59)	1	1
Refugees	422 (3.97)	2.81 (2.54 to 3.11)	1.72 (1.54 to 1.92)	315 (2.43)	0.94 (0.84 to 1.06)	0.69 (0.61 to 0.77)
**Diabetes mellitus**						
Swedish-born individuals	2062 (2.75)	1	1	3476 (3.57)	1	1
Refugees	424 (5.84)	2.16 (1.94 to 2.39)	1.30 (1.15 to 1.47)	219 (2.15)	0.60 (0.52 to 0.69)	0.47 (0.41 to 0.54)
**Diseases of the circulatory system**						
Swedish-born individuals	5683 (3.02)	1	1	6945 (2.98)	1	1
Refugees	671 (5.72)	1.92 (1.77 to 2.08)	1.33 (1.22 to 1.45)	312 (2.00)	0.67 (0.60 to 0.75)	0.52 (0.46 to 0.58)
**Diseases of the respiratory system**						
Swedish-born individuals	3781 (1.80)	1	1	4891 (2.02)	1	1
Refugees	444 (3.03)	1.70 (1.54 to 1.87)	1.08 (0.98 to 1.21)	214 (1.24)	0.61 (0.54 to 0.70)	0.47 (0.41 to 0.54)
**Diseases of the digestive system**						
Swedish-born individuals	4814 (1.71)	1	1	5987 (1.81)	1	1
Refugees	707 (3.21)	1.90 (1.76 to 2.06)	1.21 (1.11 to 1.32)	285 (1.08)	0.60 (0.53 to 0.67)	0.47 (0.42 to 0.53)
**Diseases of the musculoskeletal system**						
Swedish-born individuals	8427 (1.92)	1	1	6397 (1.23)	1	1
Refugees	1338 (4.84)	2.56 (2.42 to 2.72)	1.57 (1.47 to 1.67)	301 (0.86)	0.70 (0.63 to 0.79)	0.49 (0.44 to 0.55)
**Diseases of the nervous system**						
Swedish-born individuals	5421 (3.98)	1	1	4405 (2.42)	1	1
Refugees	548 (6.25)	1.59 (1.45 to 1.73)	1.02 (0.93 to 1.13)	165 (1.40)	0.58 (0.49 to 0.67)	0.47 (0.40 to 0.55)
**Other somatic disorders**						
Swedish-born individuals	20 443 (1.15)	1	1	19 138 (0.97)	1	1
Refugees	2690 (2.43)	2.14 (2.06 to 2.23)	1.28 (1.23 to 1.34)	866 (0.69)	0.71 (0.66 to 0.76)	0.51 (0.48 to 0.55)

*Number of individuals with the outcome during the follow-up period.

†Adjusted for sociodemographic factors, that is, sex, age, educational level, type of living area, family situation, measured on 31st of December 2009; work-related factors, that is, income from work and unemployment during 2009; and health-related factors, that is, sickness absence during 2009 and a main or secondary diagnosis of a mental disorder from inpatient or specialised outpatient healthcare 2006–2009.

#### Mental disorders

Refugees with mental disorders had in the crude model a 19-fold higher risk for disability pension compared with Swedish-born individuals without any mental disorder (table 4). The risk estimates for refugees with mental disorders were still higher compared with Swedish-born individuals with mental disorders in the full model (HR: 8.0; 95% CI: 7.7 to 8.4). The risk estimates for disability pension (compared with Swedish-born women/men without mental disorders) were slightly lower among refugee women with mental disorders (HR: 7.3; 95% CI: 6.8 to 7.8) than among refugee men with mental disorders (HR: 8.8; 95% CI: 8.3 to 9.4, figures not shown).

**Table 4:** HRs and 95% CIs of disability pension and mortality among refugees and Swedish-born individuals with or without a mental disorder, resident in Sweden in 2009; follow-up 2010–2013

	n (%)*	Crude	Model 1†	Model 2‡	Model 3§
			HR (95% CI)		
**Disability pension (n=4 058 718)**					
Swedish-born individuals, no mental disorders	9538 (0.30)	1	1	1	1
Refugees, no mental disorders	973 (0.58)	1.93 (1.81 to 2.07)	1.93 (1.80 to 2.06)	1.29 (1.21 to 1.38)	1.28 (1.19 to 1.37)
Swedish-born individuals, with mental disorders	20 282 (2.87)	9.61 (9.38 to 9.84)	8.69 (8.47 to 8.90)	7.59 (7.40 to 7.78)	6.05 (5.89 to 6.20)
Refugees, with mental disorders	2831 (5.68)	19.30 (18.51 to 20.13)	17.85 (17.09 to 18.65)	10.01 (9.56 to 10.47)	8.00 (7.65 to 8.38)
**Mortality (n=4 373 640)**	)				
Swedish-born individuals, no mental disorders	13 715 (0.42)	1	1	1	1
Refugees, no mental disorders	583 (0.34)	0.80 (0.74 to 0.87)	0.90 (0.82 to 0.97)	0.68 (0.63 to 0.74)	0.67 (0.62 to 0.73)
Swedish-born individuals, with mental disorders	16 326 (1.82)	4.31 (4.21 to 4.40)	3.31 (3.23 to 3.39)	2.65 (2.58 to 2.71)	2.23 (2.18 to 2.29)
Refugees, with mental disorders	727 (1.09)	2.59 (2.41 to 2.79)	2.07 (1.92 to 2.23)	1.30 (1.21 to 1.40)	1.08 (1.00 to 1.17)

*Number of individuals with the outcome during the follow-up period.

†Adjusted for sociodemographic factors, that is, sex, age, educational level, type of living area and family situation, measured on 31st of December 2009.

‡Like model 1 and additionally adjusted for work-related factors, that is, income from work and unemployment, measured during 2009.

§Like model 2 and additionally adjusted for sickness absence during 2009 and occurrence of a main or a secondary diagnosis of a somatic disorder 2006–2009 according to International Classification of Disorders version 10: A00–E99, G00–Z99.

Stratified analyses on specific mental disorders showed that refugees had a higher risk for disability pension compared with Swedish-born individuals with all specific disorders (table 5). In the full model, however, there was a substantially higher risk for refugees compared with Swedish-born individuals only for those with depressive disorders (HR: 1.3; 95% CI: 1.2 to 1.4).

**Table 5:** HRs and 95% CIs of disability pension and mortality among refugees with specific somatic disorders compared with Swedish-born individuals with similar somatic disorders, resident in Sweden in 2009; follow-up 2010–2013

	Disability pension (n=4 058 718)			Mortality (n=4 373 640)		
	n (%)*	Crude	Adjusted†	n (%)*	Crude	Adjusted†
**Anxiety**						
Swedish-born individuals	4880 (6.73)	1	1	2353 (2.19)	1	1
Refugees	538 (10.25)	1.55 (1.42 to 1.69)	1.16 (1.06 to 1.28)	104 (1.35)	0.62 (0.51 to 0.75)	0.52 (0.43 to 0.64)
**Bipolar disorder**						
Swedish-born individuals	1427 (12.7)	1	1	586 (2.82)	1	1
Refugees	60 (16.00)	1.35 (1.04 to 1.74)	1.12 (0.86 to 1.46)	6 (0.96)	0.34 (0.15 to 0.76)	0.37 (0.16 to 0.82)
**Depressive disorders**						
Swedish-born individuals	5416 (7.32)	1	1	2388 (2.27)	1	1
Refugees	809 (12.52)	1.76 (1.63 to 1.89)	1.31 (1.21 to 1.41)	144 (1.47)	0.65 (0.55 to 0.77)	0.50 (0.42 to 0.60)
**Post-traumatic stress disorder**						
Swedish-born individuals	341 (12.17)	1	1	95 (1.87)	1	1
Refugees	439 (15.01)	1.25 (1.09 to 1.44)	1.03 (0.87 to 1.23)	54 (1.10)	0.59 (0.42 to 0.83)	0.37 (0.25 to 0.54)
**Other stress disorders**						
Swedish-born individuals	2160 (6.00)	1	1	958 (1.97)	1	1
Refugees	364 (8.87)	1.51 (1.35 to 1.68)	1.15 (1.02 to 1.29)	53 (0.98)	0.50 (0.38 to 0.66)	0.41 (0.31 to 0.55)
**Other mental disorders**						
Swedish-born individuals	7176 (7.35)	1	1	6674 (4.08)	1	1
Refugees	581 (10.46)	1.45 (1.33 to 1.58)	1.17 (1.07 to 1.28)	191 (2.13)	0.52 (0.45 to 0.60)	0.51 (0.44 to 0.59)

*Number of individuals with the outcome during the follow-up period.

†Adjusted for sociodemographic factors, that is, sex, age, educational level, type of living area, family situation, measured on 31st of December 2009; work-related factors, that is, income from work and unemployment during 2009; and health-related factors, that is, sickness absence during 2009 and a main or secondary diagnosis of a somatic disorder from inpatient or specialised outpatient healthcare 2006–2009 according to International Classification of Disorders version 10: A00–E99, G00–Z99.

### Mortality

#### Somatic disorders

In the crude model, refugees with somatic disorders had an around 60% higher risk of mortality compared with Swedish-born individuals without somatic disorders (table 2). Refugees with somatic disorders had, however, a slightly lower risk estimate of mortality compared with Swedish-born individuals with somatic disorders. In the full model, refugees with somatic disorders had lower risk estimates for mortality (HR: 0.9; 95% CI: 0.9 to 1.0) compared with Swedish-born individuals with somatic disorders (HR: 1.7; 95% CI: 1.7 to 1.8), and noticeably lower risk than Swedish-born individuals without somatic disorders (reference group). No significant sex differences in these associations were found.

With regard to specific somatic disorders, refugees had, compared with Swedish-born individuals with similar disorders, a significantly lower risk of mortality in all the subgroups in the full model (HR range: 0.5–0.7; table 3). The estimates closest to those of the Swedish-born individuals were for neoplasms.

#### Mental disorders

In the crude model, refugees with mental disorders had a more than doubled risk of mortality compared with Swedish-born individuals without mental disorders, but a markedly lower risk estimate of mortality compared with Swedish-born individuals with mental disorders (table 4). Also in the full model, refugees with mental disorders had considerably lower risk estimates for mortality (HR: 1.1; 95% CI: 1.0 to 1.2) compared with Swedish-born individuals with mental disorders (HR: 2.2; 95% CI: 2.2 to 2.3), and hence an equal risk compared with Swedish-born individuals without mental disorders (reference group). The risk estimates for mortality (compared with Swedish-born women/men without mental disorders) were lower among refugee women with mental disorders (HR: 0.8; 95% CI: 0.7 to 0.9) than among refugee men with mental disorders (HR: 1.3; 95% CI: 1.2 to 1.4, figures not shown).

With regard to specific mental disorders, refugees had, compared with Swedish-born individuals with similar disorders, as also shown regarding the somatic disorders, a markedly lower risk of mortality during the follow-up in all the subgroups (HR range: 0.4–0.5; table 5).

## Discussion

### Main findings

Refugees had to a higher extent both mental (except for bipolar disorder) and somatic (except for neoplasm) morbidity compared with Swedish-born individuals. Additionally, refugees with specific somatic/mental disorders, especially neoplasms, diseases in the musculoskeletal system and depressive disorders had higher risk estimates of disability pension compared with Swedish-born individuals with similar disorders. Refugees with all subgroups of specific somatic and mental disorders had lower, mainly around half the risk of mortality compared with Swedish-born individuals with similar disorders.

### Disability pension

We found a considerably higher risk of disability pension among refugees with specific disorders compared with Swedish-born individuals with the same disorders. With regard to somatic disorders, this is also in consensus with previous scientific knowledge on immigrants in general.^26 27^ Especially among immigrants from low-income countries, this has been explained by poor working conditions, for example, work in unhealthy environments or irregular working hours.^26–28^ In this study, refugees were found to have a higher prevalence of most subgroups of somatic/mental disorders compared with Swedish-born individuals. When comparing refugees with mental/somatic disorders with Swedish-born with similar disorders, refugees might have a higher work incapacity related to these disorders than Swedish-born individuals as they might have sought healthcare at a later stage due to the known restrictions in access to care for refugees.^12^ Moreover, more pronounced stigma of mental disorders among refugees and lack of competence in transcultural medicine of the treating physician might result in incorrect diagnostics and subsequently treatment and prognosis. As an example, musculoskeletal disorders among refugees might, to a higher extent, reflect somatisation or symptoms of mental disorders.^17 29–32^ The analyses also revealed that comorbid mental disorders explained a part of the higher risk for disability pension among refugees with somatic disorders compared with Swedish-born individuals with somatic disorders. Comorbid disorders might therefore give refugees a higher rate of work incapacity compared with Swedish-born individuals. In order to better understand this phenomenon, studies that assess the interaction of specific mental and somatic comorbidities in the pathways to disability pension in refugees are highly warranted.

Other reasons for why refugees may encounter obstacles in establishing themselves on the labour market, which in turn may eventually lead to disability pension, may be low employability, due to existing structural barriers such as discrimination and lack of skills with regard to the demands on the labour market in the host country.^33^ Also, low health literacy and lower help-seeking behaviour among refugees, resulting in a non-optimal treatment, may be explanations to the higher risk of disability pension among refugees.^10 34^ As low educational level is more prevalent among refugees, low employability leading to marginalisation at the labour market might be another explanation to higher levels of disability pension among refugees.^2^ In addition, the design of the social security system where sickness absence cannot be granted without previous work may promote a higher level of disability pension among refugees, as also those without gainful employment are eligible to such pension. Although many factors may interact and start a downward spiral of deteriorating health and social marginalisation, which eventually might lead to premature exit from the labour market, also specific somatic and mental disorders may be key drivers in this regard.

This study is one of the first to investigate the association between specific somatic and mental disorders and subsequent disability pension among refugees. Findings do not, however, differ considerably compared with earlier studies on immigrants in general.^27 35^ This might suggest that refugee-specific risk factors like traumatic life experiences do not contribute considerably to the disabling process of becoming work incapacitated. Still, the pathways to disability pension in refugees seem to be effected by the poor labour market attachment in this group, as controlling for information on income from work had a strong effect on the risk estimates. This effect might to some extent be diagnosis specific as the estimates related to some diagnoses regarding subsequent disability pension yielded low or non-significant differences between refugees and Swedish-born individuals.

### Mortality

Despite higher prevalence of both somatic and mental morbidity, as well as high levels of disability pension, refugees with specific disorders had around half the risk of mortality during the follow-up period compared with Swedish-born individuals with the same disorders. Similar findings have been shown for immigrants in general and researchers have been puzzled by this paradox.^21 23 36^ We could now replicate these findings for refugees with several specific somatic and mental disorders. Several possible explanations have been suggested, for example, a positive health selection of immigrants, that unhealthy immigrants remigrate (salmon bias), cultural features such as better food habits, but also pure flaws in the data collection.^21 23^ A study tested all these hypotheses and concluded that the most plausible explanation is that the immigrant population seems to be highly selected due to the fact that many foreign-born with lethal disorders or vulnerabilities may already have died in their home country or during the flight or that they have not been able to make the journey due to health reasons.^23^ In this study, despite of reports on high levels of exposure to traumatic life experiences among refugees,^37^ we found a lower risk of mortality in refugees, which is in line with previous findings. However, asylum seekers do not seem to have a mortality advantage compared with the host population,^10 36^ thus the health selection process seems to involve the whole process from living in the home country until residence permit in the host country is granted.

The risk estimates for specific disorders in relation to subsequent mortality did not differ strongly with diagnoses. Moreover, risk estimates were only marginally affected when controlling for the various covariates. This suggests that there are no major differences in the risk for and in the pathways to preterm mortality for specific diagnoses among refugees compared with Swedish-born individuals. Moreover, in contrast to reported findings on lower treatment rates for refugees,^9 10 38^ our findings do not suggest major disadvantages for refugees in receiving adequate healthcare after a diagnosis.

### Strengths and limitations

The prospective cohort design with several years of longitudinal observation and use of high-quality population-based Swedish nationwide register data^39^ can be regarded as the main strengths of this study. Register data on the working-age population of Sweden was included from different sources and thereby we avoided recall and selection bias. Moreover, there was no loss to follow-up and all data on morbidity were based on physician-based diagnoses—that is, it was not based on self-reports. We also had the opportunity to include a wide range of potential confounders derived from different registers leading to results with better validity.

Also limitations of this study should be mentioned. Our data on morbidity were based on specialised care, and data on primary healthcare visits were not available. For this reason, we most likely had a selection of cases with higher medical severity. This might have led to an overestimation of the risk for disability pension or mortality among persons with specific disorders. The quality of the Swedish NPR regarding the specific diagnoses has been shown to be good,^40^ thus, misclassification due to exposure is unlikely.

## Conclusions

Refugees have a higher prevalence of most somatic and mental disorders compared with Swedish-born, and refugees with specific somatic/mental disorders have a higher risk of disability pension, but a lower risk of mortality compared with Swedish-born with these somatic/mental disorders. Possible explanations of this discrepancy include the effect of a positive health selection among refugees who resettle in Sweden. The higher risk of disability pension among refugees may hence have other causes than worse health status among refugees.

**What is already known on this subject**

- Migrant subgroups have in general been reported to have a higher prevalence of somatic and mental disorders, as well as a higher risk of disability pension compared with Swedish-born individuals.

**What this study adds**

- We now know that refugees have a higher prevalence of several subgroups of somatic and mental disorders compared with Swedish-born and that refugees with somatic/mental disorders have a considerably higher risk of a permanent exit from the labour market, by granting of disability pension, compared with Swedish-born with these disorders. Despite this, refugees with somatic/mental disorders had a significantly lower risk of mortality during the follow-up period compared with Swedish-born individuals with similar disorders. The higher risk of disability pension among refugees may hence have other causes than worse health status among refugees, and efforts to include refugees into the labour market should be prioritised.

## Data Availability

Data may be obtained from a third party and are not publicly available. Data supporting this study cannot be shared.
